# Teaching Slow Clinical Observation Through Art: A Museum-Based Innovation in Undergraduate Nursing Education

**DOI:** 10.5334/pme.2891

**Published:** 2026-09-25

**Authors:** Álvaro Carmona Pestaña, Jenifer Santos García, Patricia Ferrero Sereno

**Affiliations:** 1 Departamento de Biociencias, Facultad de Ciencias de la Salud, Universidad Loyola Andalucía, Avda. de las Universidades s/n, Dos Hermanas, 41704 Sevilla, Spain; 2 Grupo de Investigación Nurse-In, Facultad de Ciencias Biomédicas y de la Salud, Universidad Alfonso X el Sabio, Villanueva de la Cañada, Spain

## Abstract

**Background & Need for Innovation:**

Health professions education needs learning experiences that help students practise careful observation, evidence-based description, interpretive reasoning and attention to the human dimensions of illness. Undergraduate nursing education still needs clearly described, theory-informed and transferable museum-based models that move beyond cultural enrichment.

**Goal of Innovation:**

We designed a curriculum-embedded museum-based innovation for first-year nursing students. Its central contribution is a transferable sequence for slow, deliberate and dialogic observation: preparation, slow looking, evidence-based description, facilitated interpretation, humanistic reflection and clinical transfer.

**Steps taken for Development and Implementation of innovation:**

The activity was delivered at the Museo Nacional del Prado across two academic years and was informed by visual thinking approaches, experiential learning and reflective practice. Students engaged with selected artworks as complex visual and human situations rather than diagnostic riddles.

**Evaluation of Innovation:**

We used anonymous pre- and post-activity questionnaires across two cohorts, supported by open-ended responses, faculty field notes and voluntary student reflections. A total of 874 valid questionnaires were analysed as unmatched group-level responses. Students reported higher post-activity scores in perceived knowledge, observational skills and educational value, with smaller differences in empathy. Descriptive qualitative content analysis suggested that students valued close looking, art-medicine integration and clinical relevance.

**Critical Reflection on your process:**

The evaluation supports feasibility, acceptability and implementation stability, not objective improvement in clinical performance. Key lessons concern how to design and facilitate museum-based learning: preserve the slow observation sequence, avoid reducing artworks to diagnoses, and pair humanistic reflection with transfer to nursing practice.

## Background & Need for Innovation

Learning to observe is a core but often under-explicit component of health professions education. Nursing students are expected to notice subtle visual cues, describe them accurately, interpret them in context and integrate them with patients’ narratives and clinical information. However, these abilities are not only technical. They also require tolerance for ambiguity, attention to bodily difference, sensitivity to suffering and awareness that clinical meaning is shaped by context. Medical humanities have been proposed as a way of complementing biomedical training with interpretive, reflective and humanistic forms of learning [1]. Traditional classroom-based teaching may introduce these ideas conceptually, but it offers limited opportunities for students to practise slow, deliberate and dialogic observation before entering clinical environments.

Visual arts-based education has been proposed as one way to address this challenge. Previous work in medical and health professions education suggests that structured engagement with artworks can support visual literacy, observational accuracy, reflective thinking, empathy and tolerance for ambiguity [2]. In nursing education, reviews suggest potential benefits for knowledge, skills, attitudes and professional formation, while also highlighting heterogeneity in design, outcomes and reporting [3,4]. Similar approaches in nursing have suggested benefits for observational accuracy and assessment skills [5].

Museum-based learning is particularly useful because artworks provide complex and unfamiliar visual material that can be examined without the immediate pressure of clinical decision-making. In a museum, learners can practise describing before diagnosing, considering alternative interpretations and discussing how social, historical and cultural contexts shape what is seen. Recent work in *Perspectives on Medical Education* has also highlighted the need to attend not only to whether museum-based education is valuable, but to how such programmes are structured, facilitated, evaluated and adapted across settings [6,7].

Our innovation adds to this literature by offering a curriculum-embedded, nursing-focused model centered on slow, deliberate and dialogic observation. The distinctive contribution is not that art was used in health professions education, which is already well established, but that a museum visit was transformed into a pedagogical sequence that can be described, repeated and adapted: preparation, slow observation, evidence-based description, facilitated interpretation, humanistic reflection and clinical transfer. We therefore sought to move beyond a one-off cultural enrichment activity and develop a structured learning design for first-year nursing students, a group for whom early practice in observation, uncertainty and patient-centered interpretation is particularly relevant.

## Goal of Innovation

The goal of this innovation was to design, implement and evaluate a structured museum-based art observation activity for first-year undergraduate nursing students. The activity was intended to use artworks as complex visual and human situations through which students could practise slow observation, careful description, interpretive reasoning and humanistic reflection.

The innovation operated at four complementary levels. At the system level, it aimed to embed museum-based education within a formal undergraduate nursing curriculum rather than offering it as an optional extracurricular experience. At the teaching level, it introduced a facilitation approach that created space for experimentation, dialogue and uncertainty rather than privileging binary notions of correctness. At the learning level, it aimed to cultivate ways of seeing and reasoning that value slow, dialogic observation alongside the rapid problem-solving often associated with clinical work. At the professional development level, it sought to foster humanistic attention, perspective-taking and reflective practice, especially by asking students to consider the person represented in the artwork rather than treating the image as a diagnostic puzzle.

The evaluation component was designed to help readers appraise the feasibility, perceived educational value and practical transferability of the innovation. We therefore examined group-level differences in students’ perceived knowledge, observational skills, empathy and educational value, and complemented these data with descriptive qualitative content analysis of open-ended responses and implementation reflections.

## Steps taken for Development and Implementation of innovation

The innovation was developed as a curriculum-embedded learning activity within a first-year undergraduate nursing course. Its design was informed by three complementary educational principles: visual thinking, experiential learning and reflective practice. Visual Thinking Strategies and related art observation approaches emphasize careful looking, evidence-based description, dialogic interpretation and openness to multiple meanings [8]. Experiential learning theory informed the use of the museum as an authentic learning environment in which students could move from concrete experience to reflective observation and conceptual integration [9]. Reflective practice informed the final phase of the activity, in which students examined not only what they saw, but how they interpreted visual information and how those interpretations might shape nursing care [10].

The development process began with the identification of artworks that could function as complex visual cases. Rather than selecting paintings only for their aesthetic or historical relevance, we used a structured bibliographic process to identify works housed in the Museo Nacional del Prado that had been previously discussed in medical humanities or clinical literature. Priority was given to artworks depicting disease, bodily difference, disability, ageing, suffering, care or historically contextualised representations of illness. The selected works and the sources supporting their pedagogical use are listed in Supplementary File 2. No images are reproduced in the manuscript or supplementary files.

**Figure 1:**
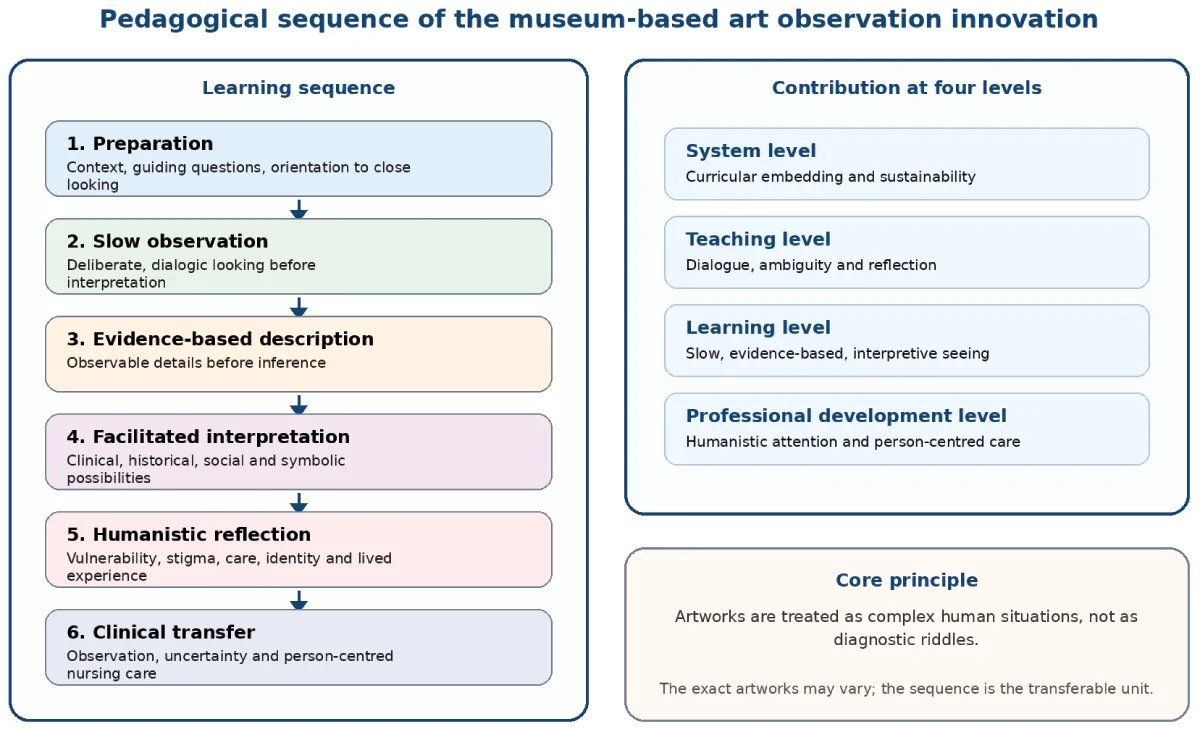
Pedagogical sequence of the museum-based art observation innovation.

The activity followed a six-part pedagogical sequence (Figure 1). First, students received preparatory material introducing the relationship between art, medicine and clinical observation. This material included brief historical context, selected examples of disease representation in art and guiding questions intended to orient students towards close looking rather than memorization of diagnoses. Second, during the museum visit, students worked in small groups guided by a faculty facilitator. They were invited to spend time with each artwork before interpretation began. This slow observation phase was intentionally designed to counter the impulse to reach for an immediate diagnostic label.

Third, students were asked to describe what they saw using observable evidence: posture, facial expression, skin appearance, bodily proportions, gestures, clothing, spatial relationships and environmental clues. Facilitators repeatedly asked students to distinguish what was visible from what was being inferred. Fourth, discussion moved into facilitated interpretation. Students considered how visual signs might relate to infectious diseases, endocrine disorders, genetic conditions, neurological phenomena, psychiatric representations or social constructions of illness, while also discussing alternative explanations and the limits of retrospective diagnosis.

Fifth, each artwork was treated as a complex human situation rather than a diagnostic riddle. Students were prompted to reflect on vulnerability, stigma, care, suffering, identity and the historical conditions under which some bodies become visible or invisible. Sixth, each visit concluded with structured group reflection focused on transfer to nursing practice: observing carefully, describing accurately, considering context, remaining open to alternative explanations and approaching patients as persons rather than as collections of signs.

It is important to distinguish reflection as a learning activity from reflection as an evaluation source. The structured group reflection was part of the intervention itself and was not audio-recorded or transcribed as formal qualitative data. Faculty field notes captured implementation issues, student engagement and observations useful for refining the activity. Voluntary student reflections submitted after the activity, together with open-ended questionnaire responses, were used as supplementary qualitative material for the evaluation.

## Evaluation of Innovation

We evaluated the innovation across two consecutive academic years using a mixed-methods educational evaluation design. First-year undergraduate nursing students were expected to complete anonymous questionnaires before and after the museum-based activity. Because no stable individual identifier was collected, responses could not be paired at the student level and were analysed as independent pre- and post-activity response groups within each cohort. In total, 874 valid questionnaires were analysed: 414 from the 2023–2024 cohort and 460 from the 2024–2025 cohort. Additional statistical detail is provided as Supplementary File 1.

The questionnaire was developed for local educational evaluation rather than as a fully validated psychometric instrument. Items were mapped conceptually to four domains aligned with the goals of the innovation: perceived knowledge, observational or visual literacy skills, empathy and perceived educational value. Selected empathy items were informed by the Jefferson empathy literature for health professions students [12,13], and selected visual observation items were inspired by visual literacy assessment work [14]. The recommendation item used a 0–10 likelihood-to-recommend format. The questionnaire was reviewed by the teaching and research team before administration, but no formal pilot validation study was conducted. For this reason, scores should be interpreted as domain-specific indicators of perceived learning and educational value, not as validated measures of competence. The item-domain mapping is provided in Supplementary File 1.

The quantitative findings are summarised in Table 1. At group level, post-activity responses were higher than pre-activity responses in perceived knowledge, observational skills and perceived educational value in both cohorts. Empathy scores also showed higher post-activity values, although differences were smaller and baseline scores were already high. Phase by cohort interaction models did not show significant interaction terms for the four main domains, suggesting a similar pattern across both academic years. These findings are best understood as signals of feasibility, perceived educational value and implementation stability, rather than evidence of objective individual-level learning gain.

**Table 1:** Summary of evaluation findings across two cohorts.


EVALUATION DOMAIN	2023–2024 COHORT	2024–2025 COHORT	INTERPRETATION FOR THE INNOVATION

Analysed questionnaires	414 total: 211 pre, 203 post	460 total: 241 pre, 219 post	Large consecutive cohorts; responses were anonymous and unmatched.

Perceived knowledge	2.67 +/– 0.57 pre; 3.63 +/– 0.38 post; g = 1.97; p < .001	2.66 +/– 0.57 pre; 3.64 +/– 0.37 post; g = 2.01; p < .001	Higher post-activity perceived knowledge scores; interpreted cautiously because of weak post-activity internal consistency.

Observational skills	3.76 +/– 0.74 pre; 4.31 +/– 0.67 post; g = 0.78; p < .001	3.78 +/– 0.78 pre; 4.29 +/– 0.68 post; g = 0.70; p < .001	Higher post-activity perceived observation scores, aligned with the central pedagogical sequence.

Empathy	4.70 +/– 0.31 pre; 4.83 +/– 0.28 post; g = 0.45; p < .001	4.75 +/– 0.35 pre; 4.83 +/– 0.28 post; g = 0.27; p < .001	Smaller group-level differences, probably influenced by high baseline scores and ceiling effects.

Perceived educational value	4.30 +/– 0.67 pre; 4.87 +/– 0.30 post; g = 1.08; p < .001	4.31 +/– 0.65 pre; 4.87 +/– 0.30 post; g = 1.07; p < .001	Students reported high perceived value and clinical relevance after the activity.

Able to identify signs/symptoms in artworks	39.0% pre; 100.0% post; p < .001	38.3% pre; 100.0% post; p < .001	Strong shift in perceived visual competence, not objective performance.

Familiarity with visual diagnostic methods	32.9% pre; 91.6% post; p < .001	31.8% pre; 92.2% post; p < .001	Students reported greater familiarity with visual diagnostic approaches.

Phase by cohort interaction	No significant interaction for the four main domains	No significant interaction for the four main domains	Similar group-level pattern across academic years, suggesting implementation stability.

Student recommendation	NPS = 83.7; mean recommendation = 9.41/10	NPS = 83.5; mean recommendation = 9.41/10	Very high acceptability.

Qualitative content categories	Specific artworks/authors; observation and visual diagnosis; art-medicine integration; teaching/communication	Same pooled pattern across available qualitative data	Students valued close looking and the art-medicine bridge; main requested improvement was longer duration.


*Note*. Values for knowledge, observational skills, empathy and perceived educational value are presented as mean +/– standard deviation. Pre- and post-activity responses were anonymous and unmatched; therefore, comparisons were analysed as independent groups within each cohort. NPS, Net Promoter Score; g, Hedges’ g.

The weak post-activity internal consistency of the three-item knowledge index was treated as an important limitation rather than as a minor technical issue. Pre-activity internal consistency was acceptable, but post-activity Cronbach’s alpha was low in both cohorts. A plausible interpretation is that after the museum experience, the knowledge items may have captured related but heterogeneous forms of perceived learning, including historical understanding, visual diagnostic familiarity and recognition of disease representation. We therefore avoid treating this domain as a robust scale and present it as a brief index of perceived knowledge.

The qualitative data collected as part of the evaluation were analysed using descriptive qualitative content analysis rather than thematic analysis [15]. Open-ended questionnaire responses were the primary qualitative source. Faculty field notes and voluntary reflections were used to contextualise implementation and interpretation, not as equally weighted outcome datasets. Coding combined deductive categories aligned with the educational goals of the innovation with inductive identification of recurrent response content. Codes were grouped into descriptive categories such as specific artworks/authors, observation and visual diagnosis, art-medicine integration, teaching/communication, longer duration and more artworks/topics. To support credibility, category definitions and illustrative excerpts were reviewed by the author team, and interpretations were checked against the quantitative findings to avoid claims unsupported by the evaluation design. Because the open-ended responses were generally brief, we did not attempt a deeper discourse-oriented or interpretive analysis.

Representative translated excerpts were added to illustrate the descriptive categories. Students described the value of slow looking by referring to “the number of hidden details in the paintings that are not visible at first sight” and by noting that, when a work was observed carefully, it could reveal signs that invited clinical discussion. Other responses linked the activity to art-medicine integration and nursing, for example valuing “the possibility of knowing what was behind each work and its relationship with nursing” and being able “to identify diseases from the paintings, especially Goya”. These excerpts are illustrative rather than evidentiary proof of learning; they show how students made sense of the activity and why the art-medicine bridge appeared meaningful to them. Representative excerpts are provided in Supplementary File 3.

The same excerpts also clarified two implementation lessons. First, some students’ language foregrounded the appeal of finding pathologies in paintings, including one response that stated that “all the paintings, if you observed them carefully, showed some pathology”. We interpret this as a useful reminder that facilitators must continually return students from diagnosis to personhood, context and uncertainty. Second, suggestions for improvement mainly concerned expansion rather than redesign, with students asking for “more time to see more works” or stating that the activity “felt quite short”.

Integration of quantitative and qualitative findings occurred at the interpretation stage. The quantitative data indicated high acceptability and higher post-activity perceived scores in domains aligned with the intervention, while the qualitative content analysis helped clarify what students appeared to value: close looking, the selected artworks, the integration of art and medicine, and the perceived relevance to clinical learning. Together, these findings support the view that the innovation was feasible and educationally meaningful to students, while leaving open the question of whether it improves objectively measured observation or clinical reasoning.

## Critical Reflection on your process

Several aspects of the innovation appeared to work particularly well. First, the museum created a low-stakes environment in which students could practise observation and interpretation without the immediate pressure of clinical decision-making. This mattered because many first-year nursing students have limited clinical exposure and may feel uncertain when asked to identify signs, symptoms or patient needs. Artworks allowed them to slow down, describe visual evidence, listen to alternative interpretations and revise first impressions before connecting what they saw with possible clinical meaning. This is consistent with previous work suggesting that art observation and Visual Thinking Strategies may support observation, critical thinking and tolerance for ambiguity in health professions education [7,8,11].

Second, the activity benefited from treating artworks as complex human situations rather than as diagnostic riddles. A purely biomedical approach to art observation risks reducing the represented person to a collection of signs. We tried to avoid this reduction by asking students to consider not only possible pathologies, but also vulnerability, stigma, historical context, social visibility and the lived experience of illness. This broader, person-centred approach to observation was especially important in nursing education, where observation is inseparable from care, communication and relational judgement. Arts-based approaches may be particularly valuable when they create space for reflective engagement with patients’ emotions, perspectives and vulnerability, although the evidence on empathy outcomes remains methodologically heterogeneous [16]. The student excerpts also sharpened our understanding of a pedagogical risk. Several students valued the possibility of identifying disease in paintings, which supports the appeal of the activity but also shows why the facilitator must prevent the session from becoming a diagnostic hunt. The educational endpoint is not the label attached to a figure in a painting; it is the disciplined movement from looking to describing, from describing to interpreting, and from interpretation back to the represented person and to nursing care.

Third, repeated implementation helped us distinguish between enthusiasm for a novel activity and a more stable signal of feasibility. The similarity of the findings across academic years suggests that the core structure of the innovation was reproducible within our curricular context. However, this apparent reproducibility within our setting should not be overstated. Reproducibility in one institution and one museum does not guarantee transferability to other settings. The Museo Nacional del Prado offered an unusually rich collection for discussing disease, bodily difference and historical representations of illness. Educators working elsewhere may need to adapt the model using local museums, digital collections or carefully selected clinical images.

For adaptation, the essential element is not access to a famous museum but preservation of the pedagogical sequence. A local museum, hospital art collection, digital archive or curated set of public-domain images could be used if the educator protects the same learning moves: preparation, slow observation, evidence-based description, facilitated interpretation, humanistic reflection and clinical transfer. The facilitator’s role is crucial. Without careful guidance, the activity can easily become either a passive cultural visit or a superficial diagnostic game. Facilitators need to ask for evidence, hold uncertainty open and repeatedly return discussion from diagnosis to personhood, context and care.

The main methodological limitation was the absence of stable identifiers. The same students were expected to complete pre- and post-activity questionnaires, but anonymity prevented us from verifying individual participation across both time points or estimating within-student change. In future iterations, we would use anonymous self-generated codes to preserve confidentiality while enabling paired longitudinal analysis. More generally, non-randomised educational innovations require careful reporting of context, implementation and evaluation design so readers can judge both credibility and transferability [17].

The second limitation was the reliance on self-reported outcomes. Self-assessment can capture perceived learning, confidence and meaning-making, but it should not be treated as a direct measure of competence [18,19]. This distinction between perceived learning and objectively measured competence is particularly relevant because our strongest quantitative differences concerned perceived knowledge and observational ability. Students experienced the activity as meaningful and useful, but we cannot conclude that they became objectively better clinical observers. Future evaluations should include performance-based assessments, such as structured image-description tasks, rubric-based observation exercises or OSCE-style stations requiring students to describe visual evidence before proposing interpretations. The broader visual art-based medical education literature has likewise called for stronger outcome measures and attention to psychometric quality, transfer to practice and long-term retention [20].

Empathy also requires cautious interpretation. Baseline scores were already high, leaving limited room for measurable improvement. In retrospect, a short self-report scale may have been too blunt to capture the reflective empathic learning we hoped to support. Future work should combine quantitative empathy measures with reflective writing, interviews or discourse-oriented analysis of how students talk about illness, vulnerability and patients’ perspectives before and after the intervention.

Overall, this innovation suggests that museum-based art observation can be integrated into undergraduate nursing education as a structured curricular strategy rather than as extracurricular enrichment. Its practical value lies in the disciplined way students are invited to look, describe, question, interpret and reflect. The next step is not simply to repeat the visit with more questionnaires, but to refine the model so that its humanistic and observational aims are matched by more robust longitudinal and performance-based evaluation.

## Artificial intelligence use statement

The authors used generative artificial intelligence tools to support language editing, restructuring and clarity during manuscript preparation. All intellectual content, study design, data analysis, interpretation of findings and final manuscript decisions were developed, verified and approved by the authors. No generative artificial intelligence tool was used to generate, analyse or interpret the study data.

## Third-party materials

No third-party-owned images, artworks or identifiable visual materials are reproduced in this manuscript. The manuscript and supplementary files describe the educational use of artworks without reproducing them.

## Additional Files

The additional files for this article can be found as follows:

10.5334/pme.2891.s1 Supplementary File 1. This file provides extended statistical analyses, including sample structure, item-domain mapping, main educational outcomes, phase by cohort interaction models, internal consistency and descriptive qualitative content analysis summary.

10.5334/pme.2891.s2 Supplementary File 2. This file provides a revised practical implementation and DOI-supported artwork-selection guide to support adaptation of the innovation in other contexts.

10.5334/pme.2891.s3 Supplementary File 3. This file provides representative anonymised student verbatim excerpts in the original Spanish with English translations.

## Data Availability

The datasets generated and analysed during the current study are not publicly available because they contain educational research data from student participants, but they are available from the corresponding author on reasonable request and subject to institutional approval.
