# Supplementary material for: Teaching Slow Clinical Observation Through Art: A Museum-Based Innovation in Undergraduate Nursing Education
Source: Perspect Med Educ. 2026 Sep 25;15(1):871–9. doi: 10.5334/pme.2891 (PMC13614244; doi:10.5334/pme.2891)
Supplement: Supplementary file 1 [file pme-15-1-2891-s1.pdf]

Extended statistical analysis and evaluation results for "Teaching Slow Clinical Observation Through Art: A Museum-Based Innovation in Undergraduate Nursing Education"

Purpose of this supplementary file

This supplementary file provides additional methodological and statistical detail to support editorial and peer review of the Show and Tell manuscript. The main manuscript deliberately foregrounds the development, implementation and transferability of the educational innovation; this file preserves the extended evaluation details so that reviewers can appraise the evidence base without shifting the main article back towards a conventional effectiveness study.

Evaluation design

The innovation was evaluated using anonymous pre- and post-activity questionnaires across two consecutive cohorts of first-year undergraduate nursing students. The same students were expected to complete questionnaires at both time points, but because no stable individual identifier was collected, pre- and post-activity responses could not be matched at the student level. Comparisons were therefore analysed as independent group-level pre- and post-activity response groups within each cohort. Findings should be interpreted as perceived educational value, feasibility and implementation stability rather than objective individual-level learning gain.

Table S1. Sample structure

Cohort and phase	Questionnaires, n	Analytical status
2023-2024 pre-activity	211	Anonymous unmatched responses
2023-2024 post-activity	203	Anonymous unmatched responses
2024-2025 pre-activity	241	Anonymous unmatched responses
2024-2025 post-activity	219	Anonymous unmatched responses
Total analysed questionnaires	874	Group-level pre-post comparisons

Outcome domains and questionnaire item mapping

The questionnaire was developed for local educational evaluation and reviewed by the teaching and research team before use. It was not formally pilot validated as a psychometric instrument. Items were mapped conceptually to domains aligned with the educational goals of the innovation. Composite scores were calculated by averaging items within each domain. This mapping is provided to clarify what the scores represent and to support cautious interpretation of the findings.

Table S2. Questionnaire item-domain mapping

Domain	Items included	Conceptual basis	Interpretation and caution
Perceived knowledge (3 items)	Self-rated knowledge of signs/symptoms of infectious diseases; signs/symptoms of genetic diseases; history of medical practice.	Local educational goals related to disease representation, historical understanding and art-medicine interpretation.	Brief index of perceived knowledge only. Post-activity internal consistency was weak, so this domain should not be treated as a robust psychometric scale.
Observational / visual literacy skills (10 items)	Capacity to identify visual details in artworks; notice subtle details in expressions/postures; relate art representations to disease; associate visual images with clinical concepts; identify patterns; distinguish key elements others might overlook; interpret visual information for clinical practice; analyse complex visual details; understand historical context; perceived relevance of painting observation to diagnostic skills.	Visual literacy and art-observation approaches, including evidence-based description before interpretation.	Indicator of perceived visual observation confidence and relevance, not a performance-based test of clinical observation.
Empathy (10 items)	Importance of understanding patients' feelings; imagining how people feel in difficult situations; role of empathy in health care; listening to patients; exploring perspectives; emotional understanding and quality of care; patient point of view; considering feelings in clinical decisions; effect of one's attitude on emotional wellbeing; reflection on patient interaction.	Jefferson empathy literature and health professions empathy constructs adapted for local educational evaluation.	Indicator of perceived empathic orientation. High baseline scores created ceiling effects and limited measurable change.
Perceived educational value (items retained as domain/indicators)	Importance of visual recognition in training; value of additional resources related to visual diagnosis; interest in learning disease through art; preference for practical visual diagnosis activities; recommendation item.	Educational acceptability, perceived curricular relevance and practical transferability.	Used to evaluate feasibility and perceived value of the innovation rather than objective educational effectiveness.
Dichotomous indicators	Previous Prado visit; familiarity with art-medicine relationship; ability to identify signs/symptoms in artworks; familiarity with visual diagnostic methods.	Contextual and self-perceived competence indicators.	Useful for describing shifts in familiarity and perceived ability, not for inferring individual-level change.

Main quantitative findings

Table S3. Pre- and post-activity group-level differences in main educational outcomes

Cohort	Outcome	Pre mean ± SD	Post mean ± SD	Difference	Hedges' g	p value
2023-2024	Knowledge	2.67 ± 0.57	3.63 ± 0.38	0.96	1.97	< .001
2023-2024	Observation	3.76 ± 0.74	4.31 ± 0.67	0.55	0.78	< .001
2023-2024	Empathy	4.70 ± 0.31	4.83 ± 0.28	0.13	0.45	< .001
2023-2024	Perceived educational value	4.30 ± 0.67	4.87 ± 0.30	0.57	1.08	< .001
2024-2025	Knowledge	2.66 ± 0.57	3.64 ± 0.37	0.98	2.01	< .001
2024-2025	Observation	3.78 ± 0.78	4.29 ± 0.68	0.51	0.70	< .001
2024-2025	Empathy	4.75 ± 0.35	4.83 ± 0.28	0.08	0.27	< .001
2024-2025	Perceived educational value	4.31 ± 0.65	4.87 ± 0.30	0.55	1.07	< .001

Phase × cohort interaction models

Linear models were fitted for each composite outcome including phase, cohort and the phase × cohort interaction. The main effect of phase was significant across all four domains, while interaction terms were not significant. This supports the interpretation that the group-level pattern was similar across the two academic years, while still remaining limited by the anonymous unmatched design.

Table S4. Phase × cohort interaction models

Outcome	Post effect (2023-2024)	Post effect p	Interaction 2024-2025 vs 2023-2024	Interaction p	R^2
Knowledge	0.963147573	4.03601E-90	0.012774037	0.844849729	0.499473478
Observation	0.548584036	2.7474E-15	-0.037094618	0.70273682	0.119472592
Empathy	0.134027502	4.70015E-06	-0.049698508	0.230737146	0.032552288
Perceived educational value	0.566140593	7.82743E-29	-0.012047108	0.861574771	0.225942971

Internal consistency

Observation and empathy showed acceptable-to-excellent internal consistency across cohorts and phases. The knowledge domain showed acceptable internal consistency before the activity but weak internal consistency after the activity, suggesting that the three post-activity knowledge items may have captured related but heterogeneous forms of perceived learning. The revised manuscript therefore frames knowledge as a brief perceived-knowledge index rather than a robust scale.

Table S5. Internal consistency of study measures

Cohort	Scale	Pre alpha	Post alpha
2023-2024	Empathy_10	0.759850514	0.843747671
2023-2024	Knowledge_3	0.69668039	0.230679762
2023-2024	Observation_10	0.892285727	0.928812303
2024-2025	Empathy_10	0.850213158	0.831673601
2024-2025	Knowledge_3	0.703825965	0.217329115
2024-2025	Observation_10	0.920541908	0.929592556

Secondary outcomes and student evaluation

Table S6. Secondary indicators and student evaluation

Cohort	Measure	Pre	Post	p value
2023-2024	Able to identify signs/symptoms in artworks, %	39.0%	100.0%	< .001
2023-2024	Familiar with visual diagnostic methods, %	32.9%	91.6%	< .001
2023-2024	Interested in learning disease through art, %	99.0%	99.5%	0.582
2023-2024	Would like practical visual diagnosis activities, %	95.2%	95.5%	0.882
2023-2024	Familiarity with art-medicine relationship (1-3)	1.27	2.61	< .001
2023-2024	Net Promoter Score	-	83.7	-
2023-2024	Mean recommendation score (0-10)	-	9.41	-
2024-2025	Able to identify signs/symptoms in artworks, %	38.3%	100.0%	< .001
2024-2025	Familiar with visual diagnostic methods, %	31.8%	92.2%	< .001
2024-2025	Interested in learning disease through art, %	99.2%	99.5%	0.617
2024-2025	Would like practical visual diagnosis activities, %	95.4%	95.4%	0.998
2024-2025	Familiarity with art-medicine relationship (1-3)	1.26	2.62	< .001
2024-2025	Net Promoter Score	-	83.5	-
2024-2025	Mean recommendation score (0-10)	-	9.41	-

Descriptive qualitative content analysis summary

The qualitative evaluation used descriptive content analysis rather than interpretive thematic analysis. Open-ended questionnaire responses were the primary qualitative source. Faculty field notes and voluntary student reflections were used to contextualise implementation, not as equally weighted outcome datasets. Codes were generated both deductively from the educational goals and inductively from recurrent response content. Frequencies below are descriptive and should be read as signals of what students foregrounded, not as quantitative evidence of learning.

Table S7. Descriptive qualitative content analysis categories

Field	Descriptive category	Count	n texts	% of texts
Liked most	Specific artworks/authors	296	420	70.48%
Liked most	Observation and visual diagnosis	157	420	37.38%
Liked most	Art-medicine integration	78	420	18.57%
Liked most	Teaching/communication	51	420	12.14%
Liked most	Other	47	420	11.19%
Suggested changes	No changes	248	413	60.05%
Suggested changes	Longer duration	89	413	21.55%
Suggested changes	Other	64	413	15.50%
Suggested changes	More artworks/topics	30	413	7.26%
Suggested changes	Logistics/organisation	12	413	2.91%
Future topics	No suggestions	167	359	46.52%
Future topics	Specific artworks/authors	89	359	24.79%
Future topics	More pathologies/historical context	77	359	21.45%
Future topics	Other	63	359	17.55%
Future topics	Expand content/time	51	359	14.21%
Future topics	Neurological/mental health	6	359	1.67%

Interpretive caution

The evaluation should be interpreted as evidence of feasibility, acceptability, perceived educational value and implementation stability. It should not be interpreted as evidence of objective improvement in clinical performance. Future evaluation should include anonymous longitudinal identifiers and performance-based tasks, such as structured visual description, rubric-scored observation exercises or OSCE-style stations.
