# Supplementary material for: Teaching Slow Clinical Observation Through Art: A Museum-Based Innovation in Undergraduate Nursing Education
Source: Perspect Med Educ. 2026 Sep 25;15(1):871–9. doi: 10.5334/pme.2891 (PMC13614244; doi:10.5334/pme.2891)
Supplement: Supplementary file 2 [file pme-15-1-2891-s2.pdf]

Supplementary File 2. Revised implementation and artwork-selection guide

Teaching Slow Clinical Observation Through Art: A Museum-Based Innovation in Undergraduate Nursing Education

Purpose of this supplementary file. This supplementary file provides practical information for educators who may wish to adapt the museum-based art observation innovation. It also expands the artwork-selection information requested during peer review by listing a non-image corpus of Museo Nacional del Prado works used as pedagogical starting points for slow clinical observation, evidence-based description, facilitated interpretation and humanistic reflection.

A. Core pedagogical sequence

- **Preparation:** Students receive brief contextual material and are oriented to close observation rather than diagnostic memorisation.
- **Slow observation:** Students remain with the artwork before interpretation begins, resisting the impulse to label or diagnose immediately.
- **Evidence-based description:** Students are asked to state visible evidence before making clinical, historical or symbolic inferences.
- **Facilitated interpretation:** The facilitator invites multiple plausible readings and explicitly discusses uncertainty and alternative explanations.
- **Humanistic reflection:** The discussion returns from signs and symptoms to personhood, vulnerability, care, stigma and context.
- **Clinical transfer:** Students consider how the same sequence could inform nursing observation, clinical reasoning and patient-centred care.

B. Expanded Prado artwork corpus and DOI-based source notes

Interpretive caution. The clinical references in this table are framed as pedagogical entry points and diagnostic hypotheses, not as definitive retrospective diagnoses. The aim is to support slow, dialogic observation and evidence-based description, while avoiding reduction of artworks or represented people to diagnostic labels.

Source policy. The source column cites only journal articles with DOI identifiers. Books, the authors' own book project and non-DOI sources have been excluded from the source list. The repeated use of Grau et al. is intentional: it is a peer-reviewed article specifically devoted to signs of illness and medical procedures in works from the Museo Nacional del Prado.

No.	Artwork	Artist / date	Medical-humanistic teaching focus	Use in the observation sequence	DOI article source(s)
1	San Jerónimo en su estudio	Marinus van Reymerswaele, 1541	Cachexia, alopecia, xanthelasma, sclerodactyly and skin lesions; ageing, asceticism and visible bodily signs.	Students practise separating visible descriptors from interpretive hypotheses when multiple signs coexist in one figure.	[1,2]
2	San Francisco abrazando a un leproso	Zacarías González Velázquez, 1787	Leprosy/Hansen disease, social exclusion, touch, stigma and care.	Useful for discussing how nursing care can involve proximity to illness while preserving dignity and personhood.	[1]
3	La oración del Huerto	Juan de Flandes, 1514-1519	Possible basal cell carcinoma or visible cutaneous lesion in a secondary figure.	Encourages students to scan beyond the central subject and attend to subtle peripheral details.	[1]
4	Cristo ante Pilatos	Francisco de Osona, c. 1490	Rhinophyma, possible myxedema/goitre, facial morphology and physiognomic interpretation.	Used to discuss the risks of reading moral character into facial appearance and the importance of careful clinical language.	[1,2]
5	Menipo	Diego Velázquez, 1638	Rhinophyma/rosacea and the boundary between clinical sign, artistic realism and characterisation.	Supports discussion of visible dermatological features without collapsing portraiture into diagnosis.	[1,2]
6	Muchachos trepando a un árbol	Francisco de Goya, 1791-1792	Possible favus/tinea capitis, poverty, childhood and environmental determinants of disease.	Useful for linking visible signs to social context and public health conditions.	[1]
7	La boda	Francisco de Goya, 1791-1792	Retrognathism, possible enucleation, alopecia/tinea capitis; social satire and visible difference.	Helps students discuss how caricature, satire and clinical observation can overlap and create interpretive risk.	[1]
8	La mujer barbuda (Magdalena Ventura con su marido)	Jusepe de Ribera, 1631	Hirsutism/hyperandrogenism, gendered embodiment, lactation, family and spectacle.	Central for discussing bodily difference, clinical hypothesis, gender, dignity and historical medical	[1,12]

No.	Artwork	Artist / date	Medical-humanistic teaching focus	Use in the observation sequence	DOI article source(s)
				curiosity.	
9	Mary Ruthven, esposa de Van Dyck	Anthony van Dyck, c. 1640	Possible female androgenetic alopecia and visible hair patterns.	Allows students to identify a discrete visual feature and consider how appearance, status and pathology interact.	[1]
10	La extracción de la piedra de la locura	Hieronymus Bosch, c. 1501-1505	Trepanation-like procedure, quackery, superstition and historical interpretations of mental illness.	Used to distinguish medical procedure, fraud, metaphor and historical belief systems around madness.	[1,10,11]
11	El charlatán sacamuelas	Theodoor Rombouts, 1620-1625	Dental extraction, pain, performance of medical authority and public spectacle.	Supports discussion of technical procedure, patient suffering, power asymmetry and trust in health care.	[1]
12	La fragua de Vulcano	Diego Velázquez, 1630	Possible scoliosis, hip pathology/lameness and mythological bodily difference.	Helps students reflect on how myth, posture and musculoskeletal interpretation intersect.	[1]
13	Cristo abrazando a san Bernardo	Francisco Ribalta, 1625-1627	Possible temporal arteritis/giant cell arteritis suggested by temporal artery enlargement.	Used as an exercise in highly focused observation of one subtle anatomical detail.	[1]
14	Resurrección de Lázaro	Juan de Flandes, 1514-1519	Possible shoulder dislocation and discussion of death, seizure, movement and interpretation.	Encourages careful distinction between biblical narrative, anatomical observation and clinical speculation.	[1]
15	El bufón el Primo	Diego Velázquez, c. 1644	Achondroplasia or skeletal dysplasia; dwarfism, court representation and disability.	Supports slow observation of proportionality, hands, facial features and sitter agency.	[1,3-5]
16	Francisco Lezcano, el Niño de Vallecas	Diego Velázquez, c. 1643-1645	Dwarfism and cognitive/disability interpretations; risks of retrospective labelling.	Used to discuss respectful language, uncertainty and the difference between description and judgement.	[1,3-5]
17	Las meninas	Diego Velázquez, 1656	Short stature, possible achondroplasia/hypopituitarism in court attendants; complex scene observation.	Ideal for scanning multiple figures and resisting premature narrowing to a single diagnostic target.	[1,3-5]
18	Perejón, bufón del conde de Benavente y del gran duque de Alba	Antonio Moro, c. 1560	Possible residual hemiplegia after stroke; asymmetry, hand posture and gait inference.	Supports discussion of neurological observation through asymmetry and functional clues.	[1]
19	La curación de Tobías	Bernardo Strozzi, 1640-1644	Blindness, ocular treatment, pseudomembranes and historical ophthalmic care.	Connects observation, treatment narratives and the symbolic weight of restoring sight.	[1]
20	Las tres Gracias	Peter Paul Rubens, 1630-1635	Possible breast pathology, joint hypermobility, scoliosis/hyperlordosis and body morphology.	Used to discuss why visual diagnosis must be cautious and why apparent signs can also be artistic choices.	[1,8,9]
21	El descendimiento de la cruz	Rogier van der Weyden, c. 1435	Syncope, pallor, grief response and possible goitre/pseudogoitre.	Allows discussion of emotion, collapse, grief, thyroid visibility and the boundary between sign and convention.	[1]
22	David vencedor de Goliat	Caravaggio, c. 1600	Possible acromegaly/gigantism, visual field impairment, trauma and decapitation narrative.	Useful for integrating endocrine, neurological and traumatic interpretations while preserving uncertainty.	[1]
23	Eugenia Martínez Vallejo, desnuda (La Monstrua desnuda)	Juan Carreño de Miranda, c. 1680	Childhood obesity, Prader-Willi syndrome debate, Cushing differential and historical display of bodies.	Central for discussing diagnostic temptation, medical uncertainty, stigma and the ethics of looking.	[1,6]
24	Eugenia Martínez Vallejo, vestida (La Monstrua vestida)	Juan Carreño de Miranda, c. 1680	Same sitter in a different representational mode; clothing, status, display and medical interpretation.	Pairs with the nude version to examine how framing, clothing and context change observation.	[1,7]
25	San Roque	Jusepe de Ribera, 1631	Plague iconography, bubo, epidemic protection and the social meaning of infectious disease.	Useful for linking individual signs with epidemic culture and public health memory.	[1,13,14]
26	Esopo	Diego Velázquez, c. 1638	Possible gouty tophus and arthritic deformity; hand observation and social/historical diet.	Invites students to examine hands as clinically informative sites rather than focusing only on faces.	[1]
27	San Benito	El Greco, 1577-1579	Possible ulnar nerve palsy; hand posture and neurological inference.	Used to practise careful description of limb posture before suggesting nerve involvement.	[1]
28	El cambista y su mujer	Marinus van Reymerswaele, 1539	Possible cervical lymphadenopathy and arachnodactyly; observation of hands and neck.	Supports scanning of small anatomical features within a busy occupational scene.	[1]

Note. The table is intentionally non-image based. It identifies artworks and pedagogical uses only. Clinical labels are framed as possible interpretations reported in DOI-indexed literature, not as confirmed retrospective diagnoses.

C. Facilitation prompts

- What do you see? Please describe only visible evidence.
- What makes you say that? Which visual feature supports your interpretation?
- What alternative explanations could fit the same visual evidence?
- Which elements are clinical, historical, symbolic, social or artistic?
- How can we discuss bodily difference without reducing the represented person to a diagnosis?
- What does the artwork invite us to notice about care, stigma, vulnerability or personhood?
- How could this sequence change the way you observe a patient in a clinical setting?

D. Pitfalls and adaptation guidance

- **Do not turn the visit into a diagnostic quiz.** The diagnostic hypothesis is a route into observation and reflection, not the endpoint of the session.
- **Use cautious retrospective language.** Prefer formulations such as “may suggest”, “has been interpreted as”, “is compatible with” or “offers an opportunity to discuss”.
- **Select fewer works than the corpus contains.** A useful session may involve 5-8 works; the full corpus is a menu for adaptation, not a single-session itinerary.
- **Train facilitators around the sequence.** Facilitators should consistently delay diagnosis, ask for visual evidence and return discussion to person-centred care.
- **Adapt beyond the Prado.** Other museums, digital collections or local image archives can be used if educators preserve the same pedagogical sequence.
- **Strengthen future evaluation.** Future iterations should pair anonymous responses and add performance-based tasks such as structured image description or OSCE-style observation stations.

E. DOI article sources used in the artwork-selection table

1. Grau JJ, Bartolomé I, Garrido C, Iranzo A. Medicine in the Prado Museum, Madrid, Spain: signs of illness, and medical procedures in the art works. Med Clin (Barc). 2022;159(10):497-504. doi:10.1016/j.medcli.2022.05.015.

2. Ruiz Seco MP. Una internista en el Museo Nacional del Prado. La facies en la pintura: ¿espejo del alma? Rev Clin Esp. 2011;211(11):591-595. doi:10.1016/j.rce.2011.02.007.

3. Friedlaender GE. Art in Science: Velázquez and Dwarfism-The Art of Observation. Clin Orthop Relat Res. 2020;478(1):31-33. doi:10.1097/CORR.0000000000001076.

4. Velázquez' Dwarfs: A Profusion of Diagnoses. JAMA. 1989;262(3):349. doi:10.1001/jama.1989.03430030037015.

5. Velázquez' Dwarfs: A Profusion of Diagnoses-Reply. JAMA. 1989;262(3):350. doi:10.1001/jama.1989.03430030037017.

6. Neggers SJCMM, Ranke MB, Hochberg Z. “La Monstrua Desnuda”: an artistic textbook representation of Prader-Willi syndrome in a painting of Juan Carreño de Miranda (1680). J Endocrinol Invest. 2017;40:747-750. doi:10.1007/s40618-017-0639-5.

7. Hochberg Z. Eugenia Martínez Vallejo, “La Monstrua Vestida”. J Endocrinol Invest. 2005;28(4):380. doi:10.1007/BF03345368.

8. Grau JJ, Estapé J, Diaz-Padrón M, Romagosa V, Martin M. Breast cancer in Rubens paintings. Breast Cancer Res Treat. 2001;68:89-93. doi:10.1023/A:1017963211998.

9. Benazzi S, et al. Training the clinical eye. Rubens’ Three Graces: how many pathologies? Ital J Anat Embryol. 2017;122(1 Suppl):111. doi:10.36253/ijae-1914.

10. The Extraction of the Stone of Madness. Southwest Respiratory and Critical Care Chronicles. 2017;5(19):45. doi:10.12746/swrccc.v5i19.396.

11. La extracción de la piedra de la locura: una visión neuroquirúrgica. Acta Med Grupo Ángeles. 2021;19(1):148-151. doi:10.35366/98593.

12. El hiperandrogenismo a través de la pintura del siglo XVII. Rev Fac Med UNAM. 2025;68(1). doi:10.22201/fm.24484865e.2025.68.1.09.

13. Perciaccante A, Coralli A, Appenzeller O, Bianucci R. Saint Roch and social distancing during pandemics: lessons to be remembered. J Relig Health. 2021;60(4):2324-2330. doi:10.1007/s10943-021-01267-w.

14. Hidalgo A, et al. El arte como herramienta docente en medicina. Educ Med. 2018;19(3):187-194. doi:10.1016/j.edumed.2017.10.004.
