# Supplementary material for: Teaching Slow Clinical Observation Through Art: A Museum-Based Innovation in Undergraduate Nursing Education
Source: Perspect Med Educ. 2026 Sep 25;15(1):871–9. doi: 10.5334/pme.2891 (PMC13614244; doi:10.5334/pme.2891)
Supplement: Supplementary file 3 [file pme-15-1-2891-s3.pdf]

## Supplementary File 3. Representative student verbatim excerpts

*Teaching Slow Clinical Observation Through Art: A Museum-Based Innovation in Undergraduate Nursing Education*

This supplementary file provides selected anonymised student excerpts from the open-ended questionnaire responses used to illustrate the descriptive qualitative content analysis in the revised manuscript. The excerpts are not intended to provide a full interpretive qualitative dataset; they are representative examples of the descriptive categories reported in the manuscript. Original excerpts are in Spanish and English translations were prepared by the authors. No identifiable personal information is included.

### A. Representative excerpts

| **Descriptive category** | **Source** | **Original Spanish excerpt** | **Author translation** | **Analytic use** |
|---|---|---|---|---|
| Close looking / hidden details | 24-25 post-activity questionnaire, liked-most response | La cantidad de detalles escondidos en las pinturas que a simple vista no se ve, descubriendo así diferentes tipos de enfermedades y deformidades. | The number of hidden details in the paintings that are not visible at first sight, thereby discovering different types of diseases and deformities. | Illustrates students' attention to slow observation and visual detail. |
| Close looking / pathology as visible clue | 24-25 post-activity questionnaire, liked-most response | Que todas las pinturas, si las observabas bien, presentaban alguna patología. | That all the paintings, if you observed them carefully, showed some pathology. | Illustrates the appeal of diagnostic looking and the need for facilitation that resists overdiagnosis. |
| Art-medicine integration and nursing | 24-25 post-activity questionnaire, liked-most response | A parte de visitar diferentes cuadros, tener la posibilidad de saber qué había detrás de cada obra y la relación con la enfermería. | Beyond visiting different paintings, having the possibility of knowing what was behind each work and its relationship with nursing. | Illustrates perceived connection between artworks, hidden context and nursing education. |
| Visual diagnosis / clinical transfer | 24-25 post-activity questionnaire, liked-most response | Poder identificar a partir de las pinturas enfermedades. Sobre todo Goya. | Being able to identify diseases from the paintings, especially Goya. | Illustrates how students framed the activity as visual identification linked to specific artists. |
| Disease representation | 24-25 post-activity questionnaire, liked-most response | Como se representaban las enfermedades tan vividamente en los cuadros. | How diseases were represented so vividly in the paintings. | Illustrates the perceived vividness of disease representation in artworks. |
| Time and expansion | 24-25 post-activity questionnaire, suggested-change response | Me hubiese gustado que fuese más larga la actividad, se me hizo bastante corta. | I would have liked the activity to be longer; it felt quite short. | Illustrates the main requested improvement: longer duration. |
| Time and expansion | 24-25 post-activity questionnaire, suggested-change response | Tener más tiempo para ver más obras. | Having more time to see more works. | Illustrates demand for more artworks and longer exploration time. |
| Preparation | 24-25 post-activity questionnaire, suggested-change response | Quizás deberiamos habernos informado antes de las enfermedades y los sintomas que ibamos a ver en los cuadros. | Perhaps we should have been informed beforehand about the diseases and symptoms we were going to see in the paintings. | Illustrates a practical suggestion for strengthening pre-visit preparation. |
| Future content | 24-25 post-activity questionnaire, future-topics response | En más enfermedades de los cuadros. | More diseases in the paintings. | Illustrates students' interest in expanding pathology-related content. |
| Future content / pacing | 24-25 post-activity questionnaire, future-topics response | Si, en general en todos los cuadros, ya que se explicaron en un tiempo muy corto. | Yes, generally in all the paintings, because they were explained in a very short time. | Illustrates interest in deeper coverage and the perceived constraint of time. |

Note. Excerpts are presented as short illustrative examples only. They should not be interpreted as evidence of objective competence or individual-level change. Because questionnaires were anonymous and unmatched, quotations cannot be linked to quantitative pre-post trajectories.
